# 

**Published:** 1817-02

**Authors:** 


### CORRESPONDENCE.

Mr. Morrison's Cases of Injuries of the Head, with the appearances on dissection, are received, and shall be inserted as soon as room can be spared.

Mr. Coley's interesting paper relative to the Upas Tree, and the experiments on living animals with the poison, are thankfully received.

M. Lespagnol " on Cerebral Inflammations as determined by Affections of the Digestive Organs," is received, as are Don Matéos and Chaumeton, on the degraded State of Medicine and its Professors in Spain.

Curious instance of Cessation of Pulse in all tangible arteries for some days, with the appearances on dissection afterwards, when the patient died, will be inserted early.

Case of Injury of the Head, by R. M. is received for *Portefeuille,* as is Case of Cardiac Disease, with Dropsical Effusion, long counteracted by medicine.

Dr. M. Petit's account of *Entero-Mesenterique Fever* will shortly appear in the *Miscellaneous Department.*

A Review of Dr. Somers's work will be inserted in our next number.

Mr. Howship's Practical Observations have been received.

A pressure of Review Matter has obliged us to defer a great number of truly interesting cases received for our Portefeuille and Miscellaneous Departments, for the communication of which we desire to express our best thanks to the respective authors.—A description of the anatomical survey of the first case in our Portefeuille of last month, shall certainly appear in our March number.

☞ Authors and Publishers are requested to send Copies of new Works for Review as early as possible after their publication. They will be reviewed early in proportion.

